# Contrasting Effects of Beneficial and Pathogenic Fungal Inoculation on Rhizosphere Microbial Community Assembly, Network Properties, and Functional Contributions of Keystone Taxa in Cucumber Soil

**DOI:** 10.3390/microorganisms14071434

**Published:** 2026-06-30

**Authors:** Wenjie Zhan, Ling Li, Jixing Zeng, Qirong Shen, Min Wang, Shiwei Guo

**Affiliations:** Jiangsu Provincial Key Lab of Solid Organic Waste Utilization, Jiangsu Collaborative Innovation Center of Solid Organic Wastes, The Key Laboratory of Plant Immunity, Nanjing Agricultural University, Nanjing 211800, China; 2020203034@stu.njau.edu.cn (W.Z.); 2018203036@njau.edu.cn (L.L.); 2021203031@njau.stu.edu.cn (J.Z.); qirongshen@njau.edu.cn (Q.S.); minwang@njau.edu.cn (M.W.)

**Keywords:** *Fusarium oxysporum*, *Trichoderma guizhouense*, community assembly, co-occurrence network, keystone taxa

## Abstract

Beneficial and pathogenic fungal inoculation can substantially influence plant growth by reshaping rhizosphere microbial communities. However, how different fungal inoculants differentially affect microbial community assembly processes, co-occurrence network stability, keystone taxa distribution, and their potential associations with plant growth remains poorly understood. Cucumber was used as the model plant, and *Fusarium oxysporum* (pathogenic, Foc) and *Trichoderma guizhouense* (beneficial, Tri) were selected as inoculants. 16S rRNA and ITS2 amplicon sequencing were used to investigate the diversity, composition, assembly processes, and co-occurrence network structure of rhizosphere bacterial and fungal communities, respectively. In addition, we used Zi–Pi topological role analysis, functional prediction, Mantel tests and random forest to characterize keystone taxa and link microbial assembly, network stability to plant nutrient and biomass traits. Foc decreased bacterial diversity while Tri increased it. Tri was associated with greater microbial network connectivity and complexity, as well as network characteristics consistent with higher inferred stability, with more connector keystone taxa enriched in glycan and terpenoid metabolic functions; by contrast, Foc simplified network structure and enriched saprotrophic fungal keystones. Bacterial assembly shifted toward deterministic processes under Foc, whereas stochastic processes remained predominant in Tri and control treatments. Random forest further confirmed divergent drivers: bacterial assembly depended mostly on community composition, while fungal assembly was regulated by plant nutrients and fungal diversity. All microbial properties were tightly linked to plant biomass and nutrient accumulation. Collectively, beneficial and pathogenic fungi exert opposing influences on rhizosphere microbial organization: Tri was associated with more connected microbial communities and a greater diversity of predicted functional traits, whereas Foc strengthened environmental filtering and simplified community structure, with plant–microbe–nutrient feedbacks likely contributing to rhizosphere assembly and ecosystem functionality.

## 1. Introduction

The assembly of microbial communities and the health of host plants are shaped by dynamic and intertwined interactions among the host, the microbes, and the environment [1,2,3]. Soil microorganisms play vital roles in ecosystem functioning by participating in biogeochemical cycling, facilitating the transformation of carbon, nitrogen, and phosphorus, and providing nutrients to plants through organic matter decomposition [4,5,6]. Some microorganisms establish symbiotic relationships with plants, promoting plant growth and contributing to soil structure formation and stabilization, thereby influencing physical properties such as aeration and water retention, which are essential for ecosystem stability and function [7,8]. Despite the considerable attention that scholars have devoted to the study of plant–microbiome–environment interactions, the extent of our understanding of these interactions remains limited due to the inherent complexity of the phenomena under investigation [9,10,11]. Therefore, in-depth investigation of soil microbial community structure is essential for maintaining ecosystem stability and promoting sustainable agricultural development. Among the multiple dimensions of microbial ecology, community assembly processes and co-occurrence network patterns are particularly important for understanding microbial structure and ecosystem functioning.

Microbial community assembly is governed by deterministic and stochastic processes [12,13,14]; Deterministic processes are primarily driven by environmental filtering, resource competition, and biotic interactions, whereas stochastic processes are associated with ecological drift, dispersal limitation, and random colonization events [15]. Exogenous microbial inoculation can substantially alter these processes by modifying niche occupancy, resource availability, and microbial interactions within resident communities [16,17,18]. Beneficial microorganisms may promote community restructuring through niche complementarity, metabolite exchange, and suppression of specific taxa, thereby facilitating coexistence and maintaining stochastic assembly patterns [19]. In contrast, pathogenic microorganisms may impose strong selective pressures by competing for similar niches, producing antagonistic compounds, or triggering host-mediated environmental changes, which can lead to the displacement of native taxa and restructuring of ecological niches [20,21]. In addition, microbial inoculation may influence stochastic processes by alleviating dispersal limitation through the introduction of new colonizers or amplifying ecological drift through disturbance-induced population fluctuations [20,22]. These combined effects can ultimately reshape community assembly trajectories. However, shifts in community composition and diversity alone cannot fully capture the ecological consequences of microbial inoculation, as they primarily reflect taxonomic turnover rather than changes in interspecific interactions [23,24,25].

Co-occurrence network analysis offers a powerful approach to move beyond this limitation. By quantifying topological properties of the co-occurrence network, this framework can reveal whether inoculation alters network complexity and stability—shifts that are indicative of underlying ecological processes and can ultimately reshape community assembly trajectories [26,27,28]. In particular, keystone taxa identified through Zi–Pi topological role analysis—an approach that categorizes nodes based on their within-module (Zi) and among-module (Pi) connectivity—including connectors, module hubs, and network hubs, are considered critical for maintaining network stability and ecosystem functioning, despite often representing only a small proportion of the total community [29,30]. These taxa can disproportionately influence nutrient cycling, microbial interactions, and plant health by linking different ecological modules and supporting functional redundancy [26,29]. Functional prediction of keystone taxa further provides important ecological insights beyond taxonomic composition, helping to reveal whether beneficial or pathogenic inoculation promotes microbial functions related to nutrient transformation, disease suppression, and ecological resilience. Nevertheless, how beneficial and pathogenic fungal inoculation differentially affect keystone taxa distribution, microbial network stability, and their potential functional roles in the rhizosphere remains poorly understood. However, previous studies have typically considered community assembly processes, co-occurrence networks, and keystone taxa separately, without fully integrating these components into a unified framework. As a result, it remains unclear how inoculation-driven shifts in assembly processes are linked to changes in network topology and keystone taxa organization, and how these jointly shape rhizosphere community stability and function.

Beneficial and pathogenic fungi represent contrasting plant-associated lifestyles that influence rhizosphere environments through their interactions with host plants. Beneficial fungi generally enhance plant growth and nutrient acquisition, whereas pathogenic fungi impair plant performance through infection and disease development. Although fungal species differ in multiple physiological traits, these contrasting plant–fungus interactions may impose different selective pressures on rhizosphere microbial communities and therefore provide a useful ecological framework for examining microbial assembly and network organization. Previous studies have shown that *F. oxysporum* is the pathogenic fungus causing cucumber *fusarium* wilt, leading to cucumber yield losses [31,32]. Additionally, studies have confirmed that *T. guizhouense* can expand root cell walls, thereby promoting root colonization, making it a beneficial fungus that enhances cucumber growth [33,34]. Therefore, this study used cucumber as a model plant, selected *Fusarium oxysporum* (a pathogenic fungus) and *Trichoderma guizhouense* (a beneficial fungus) as inoculants and aimed to address the aforementioned research gaps. Specifically, we investigated microbial diversity, community composition, assembly processes, co-occurrence network structure, and the ecological roles of keystone taxa identified by Zi–Pi analysis. Furthermore, we combined functional prediction, Mantel tests, and random forest modeling to evaluate the potential associations among microbial community assembly, network stability, nutrient accumulation, and plant growth performance. We hypothesized that: (1) inoculation with beneficial and pathogenic fungi could lead to differential changes in the diversity, composition, and co-occurrence network structure of cucumber rhizosphere microbial communities, with *Trichoderma guizhouense* possibly enhancing network complexity and stability, while *Fusarium oxysporum* might simplify microbial interactions; (2) *Fusarium oxysporum* might promote deterministic assembly processes as a result of intensified environmental filtering and niche competition, whereas *Trichoderma guizhouense* might maintain more stochastic assembly patterns through niche complementarity; and (3) changes in microbial assembly processes, keystone taxa distribution, and network complexity might be linked to plant nutrient accumulation and biomass production.

## 2. Materials and Methods

### 2.1. Plant Material and Growth Conditions

Seeds of cucumber (*Cucumis sativus L.*) cultivar ‘Jin Chun No. 4′, obtained from the seed collection maintained by the Jiangsu Provincial Key Laboratory of Solid Organic Waste Utilization, Nanjing Agricultural University, were surface-sterilized with 10% H_2_O_2_ for 10 min and then thoroughly rinsed with sterile water. The sterilized seeds were germinated on moist filter paper in Petri dishes at 28 °C in the dark for 2 days. Germinated seedlings were transferred to sterile quartz sand. The soil used in the pot experiment was a yellow-brown soil collected from a continuously cropped cucumber greenhouse located in Nandu Town, Liyang City, Jiangsu Province, China. When the first true leaves were fully expanded, the seedlings were transplanted into plastic pots (20 cm diameter and 18 cm height) containing 2.5 kg of soil.

### 2.2. Fungal Strains and Inoculation

Two fungal strains were used in this study: *Fusarium oxysporum f. sp. cucumerinum* (Foc) and *Trichoderma guizhouense NJAU 4742* (Tri). Both strains were provided by the Jiangsu Key Laboratory of High Technology Research on Solid Organic Waste Recycling, Nanjing Agricultural University, Nanjing, China. When the seedlings reached the 2–3 true-leaf stage (1 week after transplantation), Foc and Tri were applied by uniformly inoculating the soil with their respective spore suspensions to a final concentration of 10^7^ spores g^−1^ soil [35]. The control treatment (CK) received the same volume of sterile water without fungal inoculation. One cucumber seedling was maintained in each pot. The experiment included eight replicate pots for the control treatment (CK), nine replicate pots for the Foc treatment, and nine replicate pots for the Tri treatment.

### 2.3. Rhizosphere Soil and Plant Sample Collection

**Figure 1:**
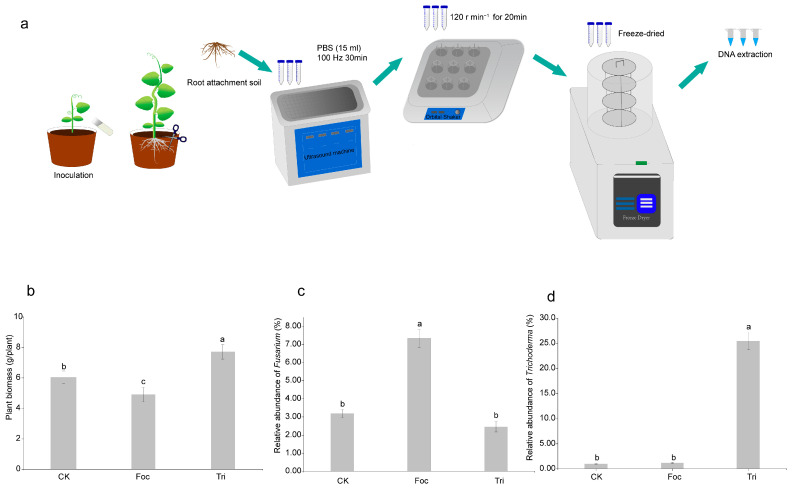
Experimental workflow and phenotypic & microbial community characteristics of cucumber plants under three treatments. (**a**) Schematic flowchart illustrating rhizosphere soil collection and pretreatment procedures for subsequent DNA extraction and high-throughput sequencing. (**b**) Shoot plant biomass (g per individual cucumber plant) across CK, Foc and Tri treatments. (**c**) Relative abundance (%) of pathogenic *Fusarium* in cucumber rhizosphere soil among different treatments. (**d**) Relative abundance (%) of biocontrol agent *Trichoderma* in cucumber rhizosphere soil under CK, Foc and Tri groups. Different lowercase letters above bars denote significant differences at *p* < 0.05 based on one-way ANOVA followed by Tukey’s HSD test.

Collect samples when the cucumber plants enter the flowering stage. The root samples were transferred to sterile centrifuge tubes (50 mL) containing 15 mL of sterile PBS solution (10 mmol L^−1^), ultrasonicated for 30 min at 100 Hz using an ultrasonic cleaner (Kunshan Ultrasonic Instruments Co., Ltd., Kunshan, China), placed on a shaking table (Shanghai Zhicheng Analytical Instrument Manufacturing Co., Ltd., Shanghai, China) at 120 r min^−1^, the Shaken at room temperature for 20 min. The washing and shaking steps were repeated twice. The soil suspension was then collected, freeze-dried, and stored in an ultra-low temperature refrigerator (Haier Biomedical Qingdao, China) at −80 °C until analysis (Figure 1). After rhizosphere soil collection, the shoots (stems and leaves) of cucumber plants were harvested, cleaned with deionized water, placed in self-sealing bags, and transported to the laboratory. The samples were initially heated at 105 °C for 30 min and then dried at 70 °C to constant weight. The dried plant samples were ground into powder and stored in self-sealing bags for nutrient analysis.

### 2.4. Determination of Plant Nutrient Content

The total nitrogen content of the plants was determined by H_2_SO_4_–H_2_O_2_ digestion followed by analysis using an AA3 continuous flow analyzer. Total phosphorus was determined by H_2_SO_4_–H_2_O_2_ digestion followed by molybdenum–antimony colorimetry. Total potassium was determined by H_2_SO_4_–H_2_O_2_ digestion followed by flame photometry. Trace elements were determined by HNO_3_–HClO_4_ digestion followed by ICP analysis.

### 2.5. DNA Extraction and Bioinformatics Analysis

DNA of rhizosphere soil samples was extracted using MoBio Soil™ DNA Separation Kit (MoBio Laboratories Inc., Carlsbad, CA, USA). Initially, 0.25 g rhizosphere soil samples were weighed according to the instructions for extraction. The amount and quality of the extracted DNA were then determined using a Nanodrop ND-2000 UV–VIS spectrophotometer (NanoDrop Technologies, Wilmington, DE, USA). The DNA is stored at −20 °C for later analysis. A total of 26 rhizosphere soil samples (CK = 8, Foc = 9, and Tri = 9) were initially subjected to amplicon sequencing. During downstream bioinformatic processing, one Foc sample was not included in the final analysis dataset. Consequently, 25 samples (CK = 8, Foc = 8, and Tri = 9) were retained for all microbial community analyses. Sequencing statistics for all samples retained for downstream analyses, including raw reads, filtered clean reads, non-chimeric reads, and Good’s coverage values, are summarized in Supplementary Table S1. Rarefaction curves based on observed zOTUs were generated separately for bacterial and fungal communities (Figure S1). Rarefaction curves approached saturation for all samples, and Good’s coverage values exceeded 0.997, indicating that the sequencing depth was sufficient to capture the vast majority of rhizosphere microbial diversity and supporting the reliability of subsequent community analyses. Barcoded primer sets 341F/805R [36] targeting the V3–V4 region of bacterial 16S rRNA genes and ITS1FI2/ITS2 [37] targeting the fungal ITS2 region were used for amplification. Libraries were sequenced on the Illumina MiSeq platform using a 2 × 250 bp paired-end strategy. Bacterial and fungal sequences were processed independently using the same bioinformatic workflow in USEARCH v11.0. Forward and reverse reads were merged using the fastq_mergepairs command with a maximum mismatch threshold (-fastq_maxdiffs) of 5. Primer sequences were trimmed prior to quality filtering, and low-quality reads were removed. After singleton filtering and chimera removal, the UNOISE3 algorithm was applied to generate zero-radius operational taxonomic units (zOTUs). Representative sequences were taxonomically assigned against the RDP database (v11.5) for bacterial 16S rRNA genes and the UNITE database (release 8.3) for fungal ITS sequences, using a confidence threshold of 0.7.

### 2.6. Analysis of Community Assembly Processes

The Beta Nearest Taxon Index (βNTI) was calculated using the Picante package in R to evaluate the relative importance of deterministic and stochastic processes in bacterial and fungal community assembly [38]. Representative bacterial 16S rRNA and fungal ITS zOTU sequences were used to construct phylogenetic trees in MEGA 11 using the maximum-likelihood method. The resulting phylogenetic trees, together with the zOTU abundance tables, were used for βNTI calculations. A null model was generated using 999 randomizations of the observed community data, including the zOTU abundance table and phylogenetic tree. The βNTI was calculated as the difference between the observed beta-Mean Nearest Taxon Distance (beta-MNTD) and the mean of the null beta-MNTD distribution, in standard deviation units. If the βNTI > 2, the observed beta-MNTD was very different from that of the stochastic simulation, suggesting that microbial community changes were mainly due to deterministic processes. A βNTI < −2 meant homogeneous selection, and a beta-NTI > 2 meant heterogeneous selection. If the |beta-NTI| < 2, the community changes were mainly due to stochastic processes. When |βNTI| < 2, an RC_Bray_ (relative contribution of Bray–Curtis dissimilarity) > 0.95 meant dispersal limitations, an RC_Bray_ < −0.95 meant homogenizing dispersal, and an |RC_Bray_| < 0.95 meant the process was not controlled by one factor. According to the null-model framework, heterogeneous selection and homogeneous selection are considered deterministic processes because they reflect environmental filtering and niche-based selection. In contrast, dispersal limitation, homogenizing dispersal, and undominated processes are classified as stochastic processes because they primarily arise from dispersal dynamics, ecological drift, and random colonization events.

### 2.7. Calculation of the Co-Occurrence Network

Microbial co-occurrence networks were constructed based on Spearman’s rank correlations using the psych package in R [39]. Correlation coefficients (ρ) and corresponding p were calculated using the corr.test function with false discovery rate (FDR) correction [40]. Only robust correlations with an absolute correlation coefficient |ρ| > 0.6 and FDR-corrected *p* < 0.05 were retained to construct the adjacency matrix, which defined the presence or absence of edges between zOTUs. The thresholds of |r| > 0.6 and FDR-adjusted *p* < 0.05 were selected based on commonly used criteria in microbial co-occurrence network analyses to reduce spurious correlations while maintaining biologically meaningful interactions. Prior to network construction, rare zOTUs were filtered to reduce noise, with bacterial zOTUs retained at a relative abundance ≥0.1% and fungal zOTUs retained at a relative abundance ≥0.02%. The adjacency matrix was used to construct an undirected network in the igraph package. Network modularity was calculated using the fast greedy algorithm, and nodes were assigned to modules based on modularity optimization. The network was visualized using Gephi (v0.9.2), where nodes represent zOTUs and were colored according to their module affiliation. Edges represent significant correlations, with red and blue lines indicating positive and negative correlations, respectively, and all edges were displayed with equal thickness.

To further assess the stability of the ecological network, species were randomly removed to simulate extinction events, and the network robustness (i.e., its resistance to node removal) was calculated. Concurrently, network vulnerability was quantified as the maximum decline in network efficiency when a single node was randomly removed, reflecting the network’s sensitivity to random species loss [41,42]. Microbial co-occurrence networks were constructed in R using the igraph package based on zOTUs correlation data, where nodes and edges represented taxa and their associations, respectively. Within-module connectivity (Zi) and participation coefficient (Pi) were calculated to determine the topological roles of each node, and nodes were classified as peripheral nodes, module hubs, connectors, or network hubs based on Zi (>2.5) and Pi (>0.62). Keystone taxa were defined as module hubs, connectors, and network hubs identified from Zi–Pi analysis, while all taxa in the network were considered the background community. Functional profiles of bacterial communities were predicted from 16S rRNA gene sequences using PICRUSt2 (v2.5.0) with default parameters, based on KEGG ortholog (KO) annotations and mapped to KEGG level-2 pathways, whereas fungal functional guilds were assigned using FUNGuild based on taxonomic annotation, and zOTUs were categorized into trophic modes (e.g., endophyte, saprotroph, and plant pathogen). Functional contributions were estimated by weighting functional annotations (KO abundance or guild assignment) with corresponding zOTUs abundance. The relative contribution of keystone taxa was calculated as the ratio of keystone-derived functional contribution to that of the whole community, with samples showing zero denominators excluded from analysis.

### 2.8. Statistical Analysis

Differences in alpha diversity (Shannon and Simpson indices) and βNTI values among treatments were assessed using the Kruskal–Wallis test implemented in the kruskal.test function in R. When significant differences were detected, pairwise comparisons were conducted using the Wilcoxon rank-sum test (wilcox.test) and resulting *p* values were adjusted using the Benjamini–Hochberg (BH) method to control the false discovery rate. Principal coordinates analysis (PCoA) was performed based on Bray–Curtis dissimilarity matrices calculated using the vegdist function (method = “bray”), and ordination was conducted using the cmdscale function to visualize differences in microbial community structure among treatments. The Mantel test was performed using the mantel function in the vegan package with 999 permutations to assess correlations between plant traits and microbial community structure. Random Forest analysis was performed using the randomForest package to evaluate the relative importance of environmental and microbial variables in shaping community assembly (βNTI). A permutation-based approach implemented in the rfPermute package was used to assess variable importance and significance, with 3000 trees (ntree = 3000) and 100 permutations (nrep = 100). Additional permutation tests (999 permutations) were conducted to evaluate predictor significance. Variable importance was determined based on the mean decrease in accuracy. All statistical analyses were performed in R (version 3.6.1), and statistical significance was determined at *p* < 0.05. Box plots and bar charts were generated using Origin 2022 (OriginLab Corporation, Northampton, MA, USA).

## 3. Result

### 3.1. Plant Growth Performance and Rhizosphere Microbial Community Structure Under Different Inoculation Treatments

Foc inoculation suppressed cucumber shoot growth, elevated rhizosphere *Fusarium* abundance and barely altered native *Trichoderma* populations. Tri application effectively alleviated *Fusarium* wilt stress, promoted plant shoot biomass accumulation, and drastically enriched biocontrol *Trichoderma* in rhizosphere soil (Figure 1b–d).

**Figure 2:**
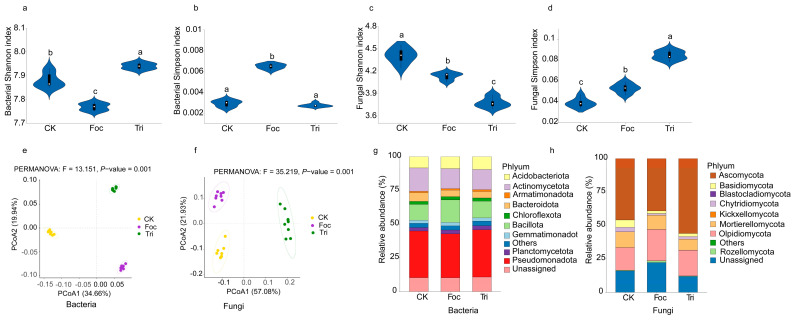
Diversity and species composition of bacteria and fungi. Alpha diversity of rhizosphere. bacterial (**a**,**b**) and fungal (**c**,**d**). Horizontal bars within boxes denote medians. Tops and bottoms of boxes represent 25th and 75th percentiles, and lines extend to the 1.5× interquartile range. Different lowercase letters indicate the significant differences (*p* < 0.05) among treatments. Principal coordinate analyses (PCoA) of weighted unifrac distances of soil bacterial (**e**) and fungal (**f**) communities. The PERMANOVA analysis showed significant differences among treatments. Relative abundance of the major phyla (top 10) in bacterial (**g**) and fungal (**h**) communities. CK, control; Foc, *Fusarium oxysporum* inoculation; Tri, *Trichoderma guizhouense* inoculation. *n* = 8 (CK), 8 (Foc), and 9 (Tri), respectively.

The Shannon index was the highest, and the Simpson index was the lowest in the Tri treatment among rhizosphere bacteria compared to the other treatments (Figure 2a,b). In contrast, the rhizosphere fungal community in the Tri treatment exhibited the lowest Shannon index and the highest Simpson index among all treatments (Figure 2c,d). The PCoA plot showed that the microbial communities clustered according to the treatments, explaining about 54.6% and 79.0% of the total variation for bacteria and fungi, respectively (Figure 2e,f).

The dominant bacterial phyla in all treatments were *Pseudomonadota*, *Actinomycetota*, and *Bacillota* (Figure 2g). The relative abundance of *Pseudomonadota* in Foc treatment was lower than that observed in the other two treatments, whereas the relative abundance of Bacillota in the Foc treatment was higher than that observed in the other two treatments (Table S2). Ascomycota and *Olpidiomycota* were identified as the predominant fungal phyla in all treatments (Figure 2h). The relative abundance of Ascomycota in the Tri treatment was higher than that observed in the other two treatments, while the relative abundance of *Olpidiomycota* was highest in the Foc treatment (Table S2).

### 3.2. Assembly of the Rhizosphere Bacterial and Fungal Communities Under Different Inoculation Treatments

**Figure 3:**
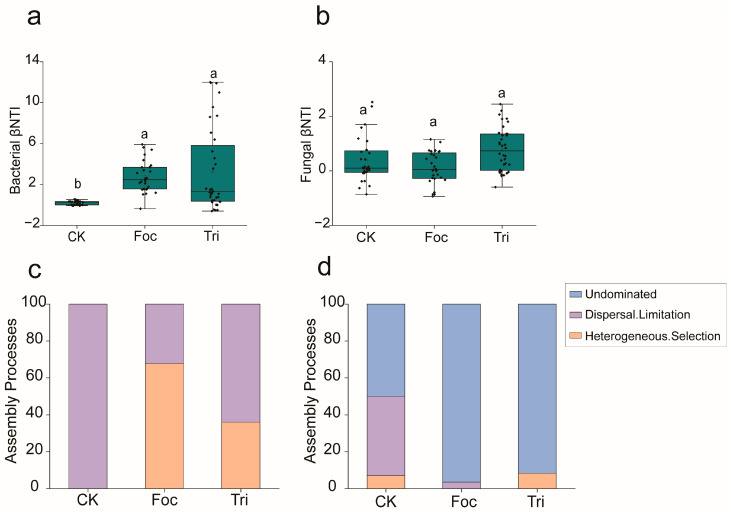
Assembly processes of the rhizosphere soil bacterial (**a**) and fungal (**b**) community in CK, Foc, and Tri treatments. Turnover fractions in the bacterial (**c**) and fungal (**d**) assemblies were dominated by stochastic (homogeneous diffusion and dispersion limited) and deterministic (heterogeneous and homogeneous selection) processes, as well as by ratios not governed by any single process (“Undominated”). CK, control; Foc, *Fusarium oxysporum* inoculation; Tri, *Trichoderma guizhouense* inoculation. Different letters indicate significant differences among treatments based on Wilcoxon rank-sum tests with Benjamini–Hochberg correction (*p* < 0.05).

We calculated the variation in deterministic and stochastic processes across the three treatments in the bacterial and fungal communities. The null model and RC_Bray_ showed that the bacterial community assembly of the CK treatment and Tri treatment was dominated by stochastic processes, while Foc treatment was dominated by deterministic processes (Figure 3a,c). The assembly of CK treatment was mainly composed of dispersal limitation, while the Tri treatment was constituted by 64% dispersal limitation and 36% heterogeneous selection. Both of these treatments were dominated by stochastic processes. The assembly of Foc treatment was composed of 68% heterogeneous selection and 32% dispersal limitation and was mainly dominated by deterministic processes (Figure 3c). While the results of the null model and RC_Bray_ analysis indicated that the fungal community was dominated by stochastic processes in all treatments (Figure 3b,d). The assembly of three treatments was mainly composed of undominated, dispersal limitation, and heterogeneous selection.

### 3.3. Co-Occurrence Networks of Bacterial Community Under Different Inoculation Treatments

**Figure 4:**
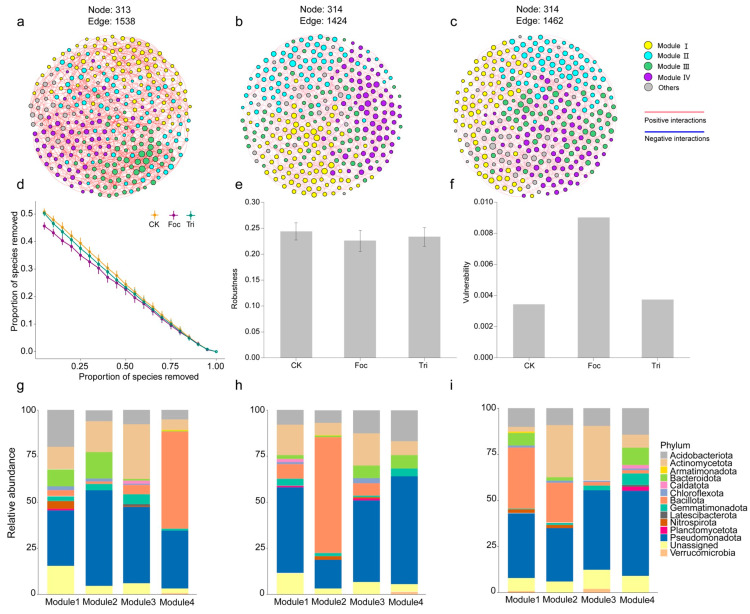
Bacterial community co-occurrence network diagram with nodes colored according to each of the four main ecological clusters (modules #1–4) of CK treatment (**a**), Foc treatment (**b**), Tri treatment (**c**). The links indicate significant co-occurrence relationships with a significant. correlation (*p* < 0.05), the blue line indicates negative relation, and the red line indicates positive relation. Network stability was assessed under simulated random species removal, including: the proportion of remaining species as a function of removed species (**d**), network robustness (**e**), and network vulnerability (**f**). Error bars are mean ± SE. The relative abundances of the four main ecological clusters at the phylum level of CK treatment (**g**), Foc treatment (**h**), Tri treatment (**i**). CK, control; Foc, *Fusarium oxysporum* inoculation; Tri, *Trichoderma guizhouense* inoculation.

Co-occurrence networks were constructed with the objective of assessing the diverse co-occurrence patterns of soil bacterial communities following inoculation of different fungal species. The correlation-based network consisted of 313 nodes and 1538 edges in the CK, 314 nodes and 1424 edges in the Foc, and 314 nodes and 1462 edges in Tri (Figure 4a–c). By removing 50% of the random nodes, the simulation of species extinction was carried out, and the robustness and vulnerability of the network were calculated. Although the bacterial network of the Foc treatment showed a trend toward lower robustness and higher vulnerability compared with the other treatments, the differences were not statistically significant (Figure 4d–f). The network modularity coefficient reflected the degree of modularity observed in the structure of the bacterial co-occurrence networks. The bar stacked chart showed the relative abundance of bacterial communities at the phylum level within the top 4 modules of the co-occurrence networks.

**Figure 5:**
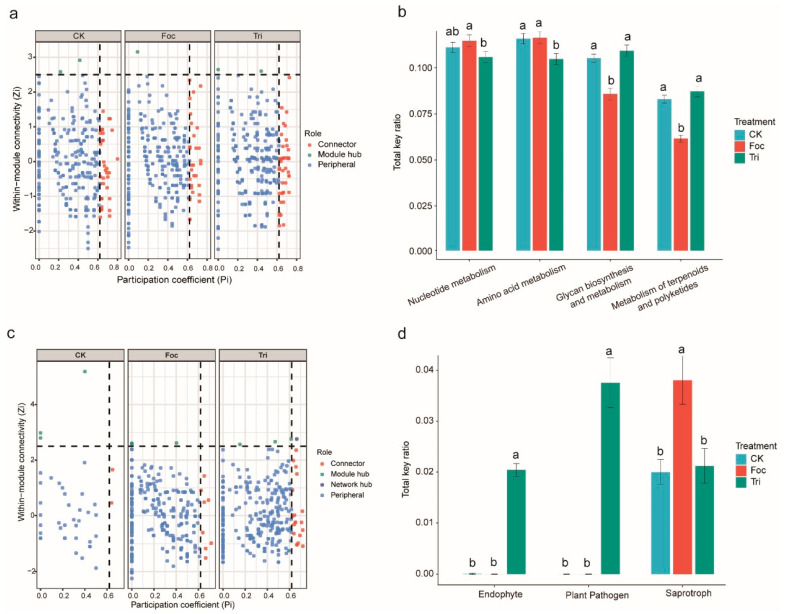
Zi–Pi topological role analysis and functional prediction of keystone taxa in bacterial and fungal co-occurrence networks under different fungal inoculation treatments. (**a**) Zi–Pi scatter plots showing node topological roles in bacterial networks under CK, Foc, and Tri treatments; (**b**) functional prediction of bacterial keystone taxa based on major metabolic pathways. (**c**) Zi–Pi scatter plots showing node topological roles in fungal networks; (**d**) functional prediction of fungal keystone taxa based on major trophic modes. Different lowercase letters indicate significant differences among treatments (*p* < 0.05). CK, control; Foc, *Fusarium oxysporum* inoculation; Tri, *Trichoderma guizhouense* inoculation.

To further elucidate the functional roles of different nodes in the bacterial co-occurrence network, node topological roles were classified using the Zi–Pi method (Table S4). The results showed that peripheral nodes overwhelmingly dominated across all treatments, accounting for 85.99–89.81% of the total nodes, whereas keystone nodes (connectors and module hubs) represented only a small proportion of the network. Notably, the Tri treatment exhibited the highest proportion of connectors (13.38%), while the Foc treatment showed the lowest proportion of keystone nodes (10.19%). The Zi–Pi scatter plot (Figure 5a) further supported this pattern, with more nodes distributed in the high-Pi region under the Tri treatment, indicating enhanced inter-module connectivity. The taxonomic identities of the dominant bacterial keystone taxa are summarized in Table S5. The major connector taxa were primarily affiliated with *Proteobacteria*, *Actinobacteria*, *Acidobacteria*, and *Bacteroidetes*. In the CK network, dominant connectors included *Chryseobacterium*, *Mesorhizobium*, *Steroidobacter*, *Thermomonas*, and *Methylophilus*, whereas the Foc network was characterized by connectors such as *Arthrobacter*, *Gp6*, *Gp1*, *Gp13*, and *Chryseobacterium*. In the Tri treatment, dominant connectors included *Pseudolabrys*, *Gp6*, *Gp1*, *Nocardioides*, and *Streptomyces*. Module hubs were represented by *Nakamurella* and an unclassified *Comamonadaceae* lineage in CK, *Betaproteobacteria* in Foc, and *Gemmatimonadetes* and *Sphaerobacter* in Tri. These taxa are generally associated with carbon turnover, nutrient cycling, organic matter decomposition, and microbial interactions. Further functional prediction analysis of keystone nodes revealed that the total key ratio in the Tri treatment was significantly higher than that in CK and Foc (Figure 5b). In particular, pathways related to glycan biosynthesis and metabolism and terpenoid metabolism were significantly enriched in the Tri treatment.

### 3.4. Co-Occurrence Networks of Fungal Community Under Different Inoculation Treatments

**Figure 6:**
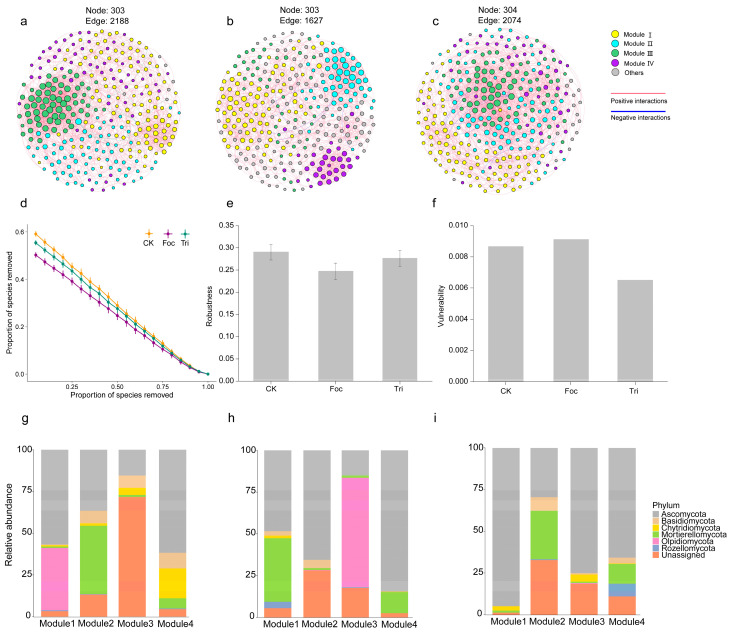
Fungal community network diagram with nodes colored according to each of the four main ecological clusters (modules #1–4) of CK treatment (**a**), Foc treatment (**b**), Tri treatment (**c**). The links indicate significant co-occurrence relationships with a significant correlation (*p* < 0.05), the blue line indicates negative relation, and the red line indicates positive relation. Network stability was assessed under simulated random species removal, including: the proportion of remaining species as a function of removed species (**d**), network robustness (**e**), and network vulnerability (**f**). Error bars are mean ± SE. The relative abundances of the four main ecological clusters at the phylum level of CK treatment (**g**), Foc treatment (**h**), and Tri treatment (**i**). CK, control; Foc, *Fusarium oxysporum* inoculation; Tri, *Trichoderma guizhouense* inoculation.

Co-occurrence networks were created to analyze the various interactions within soil fungal communities after the introduction of different fungal species. The CK correlation-based network comprised a total of 303 nodes and 2188 edges. Similarly, the Foc network comprised 303 nodes and 1627 edges, while the Tri network had 304 nodes and 2074 edges (Figure 6a–c). As with the trend of bacterial networks, the bacterial network in the Foc treatment had the lowest robustness (Figure 6d,e), and the vulnerability of the network in the Foc treatment was also higher than that in the other treatments, while the network in the Tri treatment exhibited the lowest vulnerability (Figure 6f). Similar to the pattern observed in bacterial networks, the interconnections within the Foc treatment exhibited less complexity compared to the other two treatments, suggesting that the potential interactions in the Foc treatment networks might be less stable. Across the top four modules, Ascomycota, Mortierellomycota, Chytridiomycota, and *Olpidiomycota* were identified as the predominant phyla in each treatment group. Minimal taxonomic variation in microbial phyla was observed across treatment groups, with differences primarily reflected in shifts in relative abundances (Figure 6g–i).

Similarly, Zi–Pi analysis was performed to characterize the topological roles of nodes in the fungal co-occurrence network (Table S4). The results showed that peripheral nodes also dominated the fungal networks, accounting for 92.43–96.37% of the total nodes across all treatments. However, the proportion of connectors was higher in the Tri treatment (6.25%) than in the CK (2.97%) and Foc (2.64%) treatments. In addition, network hubs were detected only in the CK and Tri treatments (0.33%) and were completely absent in the Foc treatment. The Zi–Pi scatter plot (Figure 5c) further demonstrated a more dispersed node distribution in the Tri treatment, with a greater number of high-role nodes, indicating enhanced network complexity and stability. The dominant fungal keystone taxa identified by Zi–Pi analysis are presented in Table S5. Connector taxa in the CK treatment were mainly represented by *Phialemonium*, *Aspergillus*, *Conlarium*, and *Mortierella*, while *Cephalotrichum* and *Penicillium* were important connectors in the Foc network. In the Tri treatment, dominant connectors included *Trichoderma*, *Calvatia*, *Penicillium*, *Acremonium*, and *Blastobotrys*. Module hubs were primarily affiliated with *Thielavia*, *Penicillium*, and *unclassified Ascomycota* in CK, *unclassified Ascomycota* in Foc, and *Coniochaetales* and *Sordariomycetes* in Tri. A network hub belonging to *Sordariales* was detected only in the CK treatment. These keystone fungal taxa are commonly associated with saprotrophic activity, nutrient mobilization, litter decomposition, plant-associated interactions, and biological control. Functional prediction of keystone fungal nodes (Figure 5d) revealed that the total key ratio was highest in the Tri treatment. These keystone taxa were mainly assigned to plant pathogen- and endophyte-associated functional guilds, whereas the Foc treatment showed a relatively higher proportion of saprotrophic taxa.

### 3.5. Factors Affecting the Bacterial and Fungal Community

**Figure 7:**
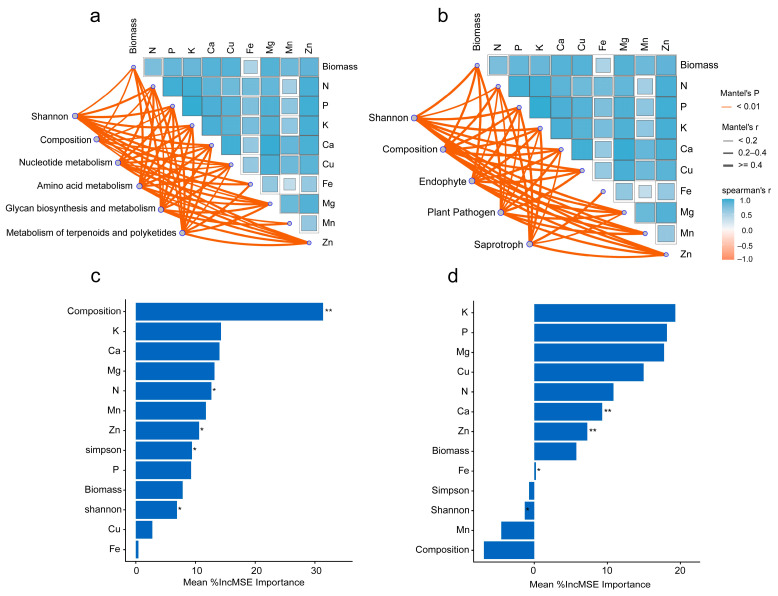
Mantel test and random forest analysis of the relationships among plant biomass, nutrient content, and rhizosphere microbial communities. Mantel test showing the correlations of biomass and plant nutrient content with microbial diversity, community composition, and keystone taxa functions in bacterial (**a**) and fungal (**b**) communities. Random forest analysis showing the relative importance of plant biomass, nutrient content, and microbial characteristics in bacterial (**c**) and fungal (**d**) community assembly. ** and * indicate significant differences at *p* < 0.01 and *p* < 0.05, respectively. Line thickness represents the strength of Mantel’s r, and color intensity indicates Spearman’s correlation coefficient. Composition represents microbial Bray–Curtis distance. N, total nitrogen; P, total phosphorus; K, total potassium; Ca, calcium; Cu, copper; Fe, iron; Mg, magnesium; Mn, manganese; Zn, zinc.

Mantel test results showed that bacterial Shannon’s diversity index, composition, and the total key ratio of keystone taxa (connectors, module hubs, and network hubs) to metabolic pathways (e.g., nucleotide metabolism, amino acid metabolism, glycan metabolism) were related to plant biomass and nutrient content (Figure 7a). For fungi, Shannon’s diversity index, composition, and the total key ratio of keystone fungi to trophic modes (e.g., endophyte, plant pathogen, saprotroph) were similarly associated with biomass and plant nutrients (Figure 7b). The plant biomass and the contents of various nutrients in the Tri treatment were significantly higher than those in the Foc treatment (Table S3). The random forest model demonstrated that bacterial composition was the most important predictor of bacterial community assembly, followed by multiple plant nutrients (e.g., K, Ca, Mg, N, Mn) and diversity indices (Figure 7c). The bacterial random forest model explained 35.2% of the variance in βNTI (OOB R^2^ = 0.352), with an OOB MSE of 2.217 and an RMSE of 1.489. For fungal community assembly, the key predictors included plant nutrients (e.g., K, P, Mg) as well as fungal Shannon and Simpson diversity indices (Figure 7d). The fungal model explained 18.9% of the variance in βNTI (OOB R^2^ = 0.189), with an OOB MSE of 0.069 and an RMSE of 0.262. These results suggest that microbial community assembly was associated with both microbial community attributes and plant nutrient status.

## 4. Discussion

### 4.1. Contrasting Effects of Beneficial and Pathogenic Fungi on Rhizosphere Microbial Diversity and Community Composition

Fungal inoculation significantly reshaped the rhizosphere microbial community of cucumber-cultivated soil, with clearly distinct effects observed between beneficial (*Trichoderma*) and pathogenic (*Fusarium oxysporum*) fungi. In this study, bacterial alpha diversity was significantly higher in the *Trichoderma*-inoculated treatment than in the *Fusarium oxysporum* and control treatments (Figure 2a,b), whereas fungal alpha diversity showed the opposite pattern, with the lowest values observed under *Trichoderma* inoculation (Figure 2c,d). These results suggest that beneficial fungal inoculation was associated with higher bacterial diversity, possibly through competitive exclusion and selective suppression of specific fungal taxa. Similar patterns have been reported previously, where *Trichoderma* inoculation increased rhizosphere bacterial diversity while reducing fungal diversity by suppressing potential pathogens and restructuring microbial interactions [43]. Although the dominant bacterial and fungal phyla remained relatively stable across treatments, their relative abundances shifted substantially, suggesting that fungal inoculation primarily altered microbial community structure rather than inducing large-scale taxonomic turnover. Such patterns are common in complex soil environments, where microbial interactions are often localized and functional shifts are more likely to occur at finer taxonomic or ecological levels rather than at broad phylum scales [12,17,44].

### 4.2. Co-Occurrence Network and Zi–Pi Analyses Reveal Differences in Microbial Network Complexity and Stability

Co-occurrence network and Zi–Pi analyses provided further insights into microbial interactions beyond conventional diversity metrics [45]. The *Trichoderma*-inoculated soil exhibited more edges, higher connectivity, greater robustness, lower vulnerability, and a higher proportion of connector nodes than the *Fusarium oxysporum*-inoculated soil, suggesting a more complex network structure and a greater potential capacity to maintain network integrity under simulated perturbations (Figure 4 and Figure 5; Table S4). Previous studies have similarly shown that higher abundance of beneficial microorganisms such as *Trichoderma* is often associated with enhanced microbial connectivity and network stability, whereas pathogen-dominated systems tend to exhibit simplified and less connected networks [46,47]. In particular, the greater number of nodes distributed in the high-Pi region under the *Trichoderma* treatment, especially in the fungal network, indicates stronger inter-module connectivity and higher ecological complexity. Higher proportions of connector nodes are generally interpreted as indicators of greater potential network stability and functional resilience. In contrast, pathogen invasion often disrupts these associations, reduces network complexity, and may decrease the inferred capacity of microbial networks to resist environmental disturbances [48]. Although correlation-based co-occurrence networks reflect statistical associations rather than direct ecological interactions and cannot establish causality, the consistent differences observed among treatments strongly suggest that fungal inoculation was closely associated with distinct patterns of microbial network organization. In the top four modules, the dominant microbial phyla were largely conserved across treatments, and the main differences were reflected in their relative abundances (Figure 4g–i and Figure 6g–i). This indicates that beneficial and pathogenic fungal inoculation may be associated with changes in patterns of bacterial co-occurrence and relative abundance [49].

### 4.3. Keystone Taxa Functional Shifts Reflected Contrasting Ecological Strategies Under Fungal Inoculation

Functional prediction of Zi–Pi keystone taxa further revealed clear ecological differences among treatments. The *Trichoderma* treatment showed a significantly higher total key ratio in both bacterial and fungal communities, indicating a greater potential contribution of keystone taxa to predicted ecosystem functions. In the bacterial network, pathways related to glycan biosynthesis and metabolism and terpenoid metabolism were significantly enriched under *Trichoderma* inoculation. Glycan biosynthesis is closely associated with microbial cell wall formation, extracellular polysaccharide production, biofilm development, and rhizosphere colonization, all of which have been associated with nutrient cycling and plant–microbe interactions in previous studies. Terpenoid metabolism is often linked to the production of antimicrobial compounds and the induction of plant defense responses [50,51]. The enrichment of these predicted pathways suggests that *Trichoderma* inoculation may increase the potential functional capacity of microbial communities associated with nutrient transformation, microbial colonization, and disease suppression. In contrast, under the *Fusarium oxysporum* treatment, fungal keystone nodes were exclusively assigned to saprotrophic fungi, suggesting reduced diversity of predicted ecological guilds within keystone taxa. The dominance of saprotrophic fungi under pathogen stress suggests that the fungal network shifted toward a basic decomposition-oriented state, which may reflect the loss of mutualistic interactions and reduced ecological complexity. Notably, the *Trichoderma* treatment restored multiple functional guilds among keystone taxa, including endophytes, plant pathogens, and saprotrophs, indicating improved functional diversity and ecological redundancy. Although the presence of plant pathogen-related taxa may appear contradictory, FUNGuild assignments are based on ecological guild annotations rather than direct measurements of pathogenic activity. Therefore, the observed enrichment indicates a higher representation of taxa assigned to the plant pathogen guild among keystone nodes. The ecological roles of these taxa may vary depending on environmental conditions and microbial interactions [52]. The reintroduction of multiple functional guilds into central network positions may be associated with higher functional diversity and ecological redundancy, which has been reported in previous studies to correlate with ecosystem stability [53,54].

### 4.4. Pathogenic Fungal Invasion Strengthened Deterministic Bacterial Community Assembly

Microbial community assembly is influenced by both deterministic and stochastic processes [55]. Deterministic assembly is predictable and primarily driven by environmental selection and biotic interactions [15], whereas stochastic assembly is influenced by unpredictable factors such as ecological drift and dispersal limitations [56,57]. In this study, the bacterial community in the *Fusarium oxysporum* treatment was dominated by deterministic processes, whereas stochastic processes remained predominant in the *Trichoderma* and control treatments (Figure 3a,c). This suggests that pathogen invasion may intensify environmental selection and strengthen deterministic assembly. Previous studies have shown that *Fusarium oxysporum* can alter the soil microenvironment by secreting toxic metabolites, changing soil pH, and intensifying competition for nutrients and spatial niches [58,59,60]. Such selective pressure may homogenize microbial communities in a predictable manner and promote deterministic assembly [61,62,63], which is consistent with our results. In contrast, beneficial fungi such as *Trichoderma* are generally considered to exert relatively moderate environmental filtering effects and often coexist with resident microbiota through niche complementarity and resource partitioning rather than intense competition [64,65]. In contrast to the bacterial response, the assembly process of the fungal community was not significantly altered by inoculation with either *Trichoderma* or *Fusarium oxysporum* (Figure 3b), which may reflect the higher niche differentiation and ecological stability of soil fungal communities [49]. It should be noted that fungal phylogenetic trees were reconstructed from ITS sequences. Although ITS is the standard barcode marker for fungal community profiling, its high sequence variability and alignment uncertainty may reduce phylogenetic resolution compared with bacterial 16S rRNA genes. Therefore, βNTI-based ecological process inference for fungal communities should be interpreted with appropriate caution. However, these possibilities remain speculative and require further investigation.

### 4.5. Microbial Assembly and Functional Traits Were Closely Linked to Plant Nutrient Accumulation and Biomass Production

Mantel and random forest analyses further revealed strong linkages among microbial community assembly, plant nutrient accumulation, and biomass production. Both bacterial and fungal Shannon diversity, community composition, and key functional groups showed significant correlations with plant nutrient contents and biomass. For bacteria, Shannon diversity and community composition were strongly associated with N, P, K, and Zn accumulation as well as biomass, while fungal diversity and composition were more strongly related to Ca, Mg, and Mn (Figure 7). These patterns suggest that bacterial and fungal communities may be differentially associated with plant nutrient status and growth performance through distinct nutrient-related pathways. Previous studies have shown that plant nutrient status strongly influences rhizosphere microbial diversity by regulating root exudation patterns and nutrient availability, while microbial communities in turn enhance plant nutrient acquisition through nutrient mobilization, mineral solubilization, and symbiotic interactions [66,67]. However, given the correlational nature of the present analyses, these patterns should be interpreted as co-variation rather than directional effects. In bacterial keystone taxa, amino acid metabolism, glycan biosynthesis, nucleotide metabolism, and terpenoid metabolism were strongly correlated with nutrient accumulation and biomass. Amino acid metabolism is closely associated with microbial growth and nutrient cycling, whereas terpenoid and polyketide metabolism is widely linked to antimicrobial compound production and plant defense responses [68,69]. In the fungal network, endophytic fungi showed strong positive correlations with biomass and nutrient accumulation, and they are widely recognized for improving host nutrient uptake and enhancing resistance to biotic and abiotic stress [70]. Because both Mantel and random forest analyses are correlation-based approaches, these relationships should be interpreted cautiously, as they likely reflect reciprocal or indirect associations among plant nutrient status, microbial community assembly, and functional profiles, rather than direct causal effects. Random forest analysis based on βNTI further showed that bacterial assembly was primarily associated with community composition and nutrient variables such as N and Zn, whereas fungal assembly was more strongly linked to Ca, Zn, Fe, and Shannon diversity. Overall, these results indicate close statistical associations among microbial assembly processes, nutrient dynamics, and plant growth performance.

### 4.6. Limitations and Future Perspectives

It should be noted that this study was conducted at a single developmental stage, which limits the ability to evaluate temporal variation and long-term stability of microbial communities. Therefore, interpretations regarding network stability and community assembly should be considered within this temporal context. In addition, the co-occurrence networks constructed in this study were based on correlation analyses and therefore represent statistical associations rather than direct biological interactions. Positive and negative correlations may arise from shared environmental preferences, indirect ecological effects, or responses to common environmental drivers. Consequently, the inferred network topology and keystone taxa should be interpreted with appropriate caution. In addition, functional annotations generated by PICRUSt2 and FUNGuild represent predictions of potential functions based on taxonomic information rather than direct measurements of gene expression, metabolic activity, or ecological function. Therefore, interpretations regarding nutrient transformation, disease suppression, functional redundancy, and ecosystem functioning should be considered cautiously and require experimental validation. Future studies incorporating longitudinal sampling across multiple plant developmental stages, combined with metabolomic and functional validation approaches, will be necessary to better elucidate the causal relationships among microbial assembly, nutrient cycling, and plant growth regulation.

## 5. Conclusions

This study demonstrates that inoculation with beneficial (*Trichoderma guizhouense*) and pathogenic (*Fusarium oxysporum*) fungi exerts contrasting effects on the cucumber rhizosphere microbiome. *Trichoderma* inoculation enhanced bacterial diversity and promoted a more interconnected microbial network with greater inferred stability, whereas *Fusarium oxysporum* reduced microbial complexity and strengthened deterministic assembly processes in bacteria. Moreover, microbial diversity, network features, and assembly processes were closely associated with plant biomass and nutrient accumulation, suggesting coordinated plant–microbe–environment feedbacks. Overall, these results highlight that fungal inoculation plays a key role in shaping rhizosphere microbial organization and associated ecosystem functions. However, given the correlation-based nature of the network analyses and the single-time-point sampling design, further longitudinal and experimental studies are needed to validate the causal mechanisms underlying these observed patterns.

## Data Availability

Relevant bacterial and fungal sequences have been uploaded to NCBI. Accession: PRJNA1296775 and PRJNA1297029.

## Supplementary Materials

The following supporting information can be downloaded at: https://www.mdpi.com/article/10.3390/microorganisms14071434/s1, Figure S1. Rarefaction curves of observed zOTUs for bacterial and fungal communities. Table S1. Sequencing quality control statistics of bacterial 16S rRNA and fungal ITS amplicons. Table S2: The relative abundances at the phylum level of the top ten bacteria and fungi in terms of ranking; Table S3: Plant biomass and plant nutrient content under different inoculation treatments; Table S4: Proportional distribution of node roles (Zi–Pi classification) in bacterial and fungal co-occurrence networks under different treatments. Table S5. Major keystone microbial taxa identified by Zi–Pi co-occurrence network analysis and their putative ecological roles.
