# Supplementary material for: Contrasting Effects of Beneficial and Pathogenic Fungal Inoculation on Rhizosphere Microbial Community Assembly, Network Properties, and Functional Contributions of Keystone Taxa in Cucumber Soil
Source: Microorganisms. 2026 Jun 30;14(7):1434. doi: 10.3390/microorganisms14071434 (PMC13413897; doi:10.3390/microorganisms14071434)
Supplement: Supplementary file 1 [file microorganisms-14-01434-s001.zip › microorganisms-4338602-supplementary.pdf]

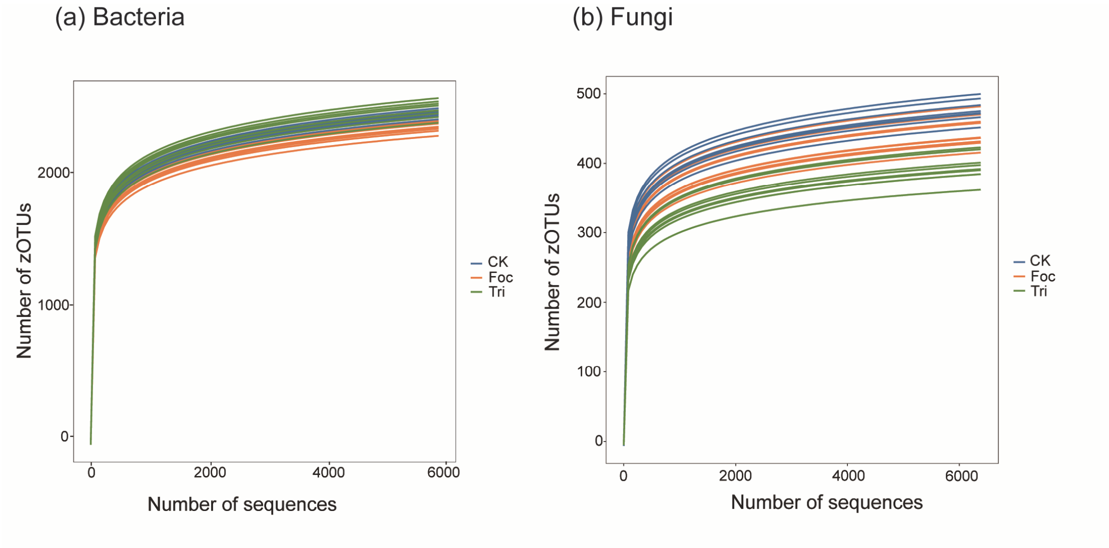

Figure S1. Rarefaction curves of observed zOTUs for bacterial and fungal communities. (a) Bacterial community rarefaction curves; (b) Fungal community rarefaction curves. The x-axis represents the number of sequences per sample, and the y-axis indicates the number of detected zOTUs. Curves are colored by treatment groups: blue = CK, orange = Foc, green = Tri. All curves reached distinct plateaus, confirming sufficient sequencing depth to recover most rhizosphere microbial zOTUs.

Table S1. Sequencing quality control statistics of bacterial 16S rRNA and fungal ITS amplicons

**Sample**	**Bacterial**				**Fungal**			
	Raw reads	Clean	Effective	Coverage	Raw reads	Clean	Effective	Coverage
CK1	90592	86231	62569	0.998224	87370	71486	68237	1.00000
CK2	88718	84747	62599	0.998429	81838	68355	65149	0.99995
CK3	97551	92967	66329	0.998276	82036	70981	68493	0.99995
CK4	91792	87437	63009	0.998532	82542	70733	67952	0.99990
CK5	92720	88653	64506	0.998498	79654	67731	65014	1.00000
CK6	91275	87005	65281	0.998515	85383	71490	67555	1.00000
CK7	89663	85834	62891	0.998651	83188	70099	66721	0.99998
CK8	89538	85206	62774	0.998668	83083	70345	66990	0.99995
Foc1	89294	85055	63808	0.998361	83077	71552	68253	0.99987
Foc2	92894	88861	65241	0.998817	79960	67707	64499	0.99998
Foc3	97490	92300	65902	0.998463	82134	69997	67100	0.99993
Foc4	88706	84711	63733	0.998907	86311	72811	67015	0.99992
Foc5	89650	85231	63973	0.998498	80343	68373	64756	0.99998
Foc6	89287	85047	62235	0.998822	81462	68541	65267	0.99998
Foc7	91527	87023	64004	0.998720	83816	71102	67468	0.99996
Foc8	90618	86608	63707	0.998429	80985	69093	66325	0.99996
Tri1	88855	84908	62024	0.998868	77481	66751	63814	1.00000
Tri2	93193	88492	65970	0.998532	78845	69514	66817	0.99998
Tri3	91330	87163	63389	0.998532	80840	71336	68766	0.99993
Tri4	96551	91963	66409	0.998190	81826	71470	68758	0.99992
Tri5	89353	84828	61878	0.998634	77474	67295	64608	0.99998
Tri6	92040	87639	64710	0.998583	82129	71177	65484	0.99998
Tri7	92156	87317	65217	0.998634	80401	70633	67500	0.99998

Tri8	95117	90632	66033	0.998395	80619	70815	67406	0.99998
Tri9	94819	90391	67386	0.997968	77883	67657	64478	1.00000

Note: Raw: total raw sequencing read pairs; Clean = sequences retained after quality trimming; Effective: final non-chimeric valid sequences for downstream community analysis; Coverage: Good's coverage index evaluating sequencing saturation. All samples exhibited Good's coverage above 0.997, indicating sufficient sequencing depth to capture most rhizosphere microbial taxa.

Table S2 The relative abundances at the phylum level of the top ten bacteria and fungi in terms of ranking

Bacteria	CK	Foc	Tri	Fungi	CK	Foc	Tri
Pseudomonadota	34.6a	32.6b	35.0a	Ascomycota	45.9b	39.1c	56.0a
Actinomycetota	17.3a	14.8b	15.0b	Olpidiomycota	16.9b	23.5a	19.3b
Bacillota	11.7c	16.7a	12.4b	Mortierellomycota	11.7b	10.6a	8.16c
Acidobacteriota	8.35c	8.89b	9.47a	Basidiomycota	5.44a	2.00b	2.32b
Bacteroidota	6.45a	4.81b	4.79b	Chytridiomycota	3.36a	1.16b	1.96ab
Planctomycetota	2.59c	2.85b	3.02a	Rozellomycota	0.48b	1.29a	0.35c
Gemmatimonadota	2.30b	2.55a	2.38b	Mucoromycota	0.05a	0.04a	0.05a
Chloroflexota	2.25a	2.37a	2.37a	Blastocladiomycota	0.01a	0.04a	0.01a
Armatimonadota	1.02c	1.11b	1.34a	Kickxellomycota	0.02a	0.02a	0.02a

Note: Different lowercase letters indicate significant differences ($p < 0.05$) in the relative abundance (%) of microorganisms at the phylum level during different treatments.

Table S3 Plant biomass and plant nutrient content under different inoculation treatments.

Treatment	Biomass	TN	TP	TK	Ca	Cu	Fe	Mg	Mn	Zn
	(g/plant)	(g/kg)	(g/kg)	(g/kg)	(g/kg)	(mg/kg)	(mg/kg)	(mg/kg)	(mg/kg)	(mg/kg)
CK	6.05±0.39a	63.74±2.22b	71.70±9.34b	79.46±10.94b	31.98±3.67b	0.29±0.07b	23.76±7.95a	64.40±7.47b	4.93±1.12b	9.37±0.94b
Foc	5.73±0.43a	31.52±4.65c	38..08±4.28c	43.10±4.07c	21.84±1.49c	0.23±0.00c	9.29±1.54b	40.95±2.85c	5.84±1.36b	4.43±0.41c
Tri	7.72±0.47b	75.87±2.50a	94.16±7.63a	103.63±5.78a	50.30±4.33a	0.44±0.03a	22.26±3.08a	107.8±15.02a	11.06±2.23a	13.42±1.44a

Note: TN: Plant total nitrogen content, TP: Plant total phosphorous content, K: Plant total potassium content, Ca: Plant calcium content, Cu: plant copper content, Fe: Plant iron content, Mg: Plant magnesium content, Mn: Plant manganese content, Zn: Plant zinc content. Different lowercase letters indicate that the average values of plant biomass and nutrient content at different treatments differ significantly (p <0.05).

Table S4. Proportional distribution of node roles (Zi–Pi classification) in bacterial and fungal co-occurrence networks under different treatments.

Treatment	Bacterial (%)			Fungal (%)			
	Connector	Module hub	Periphral	Connector	Module hub	Network hub	Periphral

CK	11.18	0.64	88.18	2.97	1.32	0.33	95.38
Foc	9.87	0.32	89.81	2.64	0.99	0	96.37
Tri	13.38	0.64	85.99	6.25	0.99	0.33	92.43

Note: Connector, module hub, and network hub are considered key nodes based on Zi–Pi analysis, while peripheral nodes represent less connected taxa.

Table S5. Major keystone microbial taxa identified by Zi–Pi co-occurrence network a nalysis and their putative ecological roles.

Kingdom	Treatment	Keystone type	Taxon	Phylum	Putative role
Bacteria	CK	Connector	*Chryseobacteriu*	*Bacteroidetes*	OM degradation
Bacteria	CK	Connector	*Mesorhizobium*	*Proteobacteria*	N fixation
Bacteria	CK	Connector	*Steroidobacter*	*Proteobacteria*	C transformation
Bacteria	CK	Connector	*Thermomonas*	*Proteobacteria*	C cycling
Bacteria	CK	Connector	*Methylophilus*	*Proteobacteria*	C1 metabolism
Bacteria	CK	Module hub	*Comamonadacea*	*Proteobacteria*	Nutrient cycling
Bacteria	CK	Module hub	*Nakamurella*	*Actinobacteria*	OM decomposition
Bacteria	Foc	Connector	*Arthrobacter*	*Actinobacteria*	Stress tolerance
Bacteria	Foc	Connector	*Gp6*	*Acidobacteria*	C cycling
Bacteria	Foc	Connector	*Gp1*	*Acidobacteria*	C cycling
Bacteria	Foc	Connector	*Gp13*	*Acidobacteria*	OM turnover
Bacteria	Foc	Connector	*Chryseobacteriu*	*Bacteroidetes*	OM degradation
Bacteria	Foc	Module hub	*Betaproteobacteri*	*Proteobacteria*	Nutrient cycling
Bacteria	Tri	Connector	*Pseudolabrys*	*Proteobacteria*	Nutrient cycling
Bacteria	Tri	Connector	*Gp6*	*Acidobacteria*	C cycling
Bacteria	Tri	Connector	*Gp1*	*Acidobacteria*	C cycling
Bacteria	Tri	Connector	*Nocardioides*	*Actinobacteria*	OM decomposition
Bacteria	Tri	Connector	*Streptomyces*	*Actinobacteria*	Biocontrol
Bacteria	Tri	Module hub	*Gemmatimonadet*	*Gemmatimonadete*	Nutrient cycling
Bacteria	Tri	Module hub	*Sphaerobacter*	*Chloroflexi*	OM decomposition
Fungi	CK	Connector	*Phialemonium*	*Ascomycota*	Lignocellulose degradation
Fungi	CK	Connector	*Aspergillus*	*Ascomycota*	OM mineralization
Fungi	CK	Connector	*Conlarium*	*Ascomycota*	Litter decomposition
Fungi	CK	Connector	*Mortierella*	*Mortierellomycota*	P solubilization & biocontrol
Fungi	CK	Module hub	*Thielavia*	*Ascomycota*	Cellulose degradation
Fungi	CK	Module hub	*Penicillium*	*Ascomycota*	Nutrient mobilization & biocontrol
Fungi	CK	Module hub	*Ascomycota*	*Ascomycota*	General saprotrophy
Fungi	CK	Network hub	*Sordariales*	*Ascomycota*	Multi-functional nutrient cycling
Fungi	Foc	Connector	*Cephalotrichum*	*Ascomycota*	Stress-resistant saprotrophy
Fungi	Foc	Connector	*Penicillium*	*Ascomycota*	Nutrient mobilization & biocontrol
Fungi	Foc	Module hub	*Ascomycota*	*Ascomycota*	General saprotrophy
Fungi	Tri	Connector	*Trichoderma*	*Ascomycota*	Mycoparasitism & biocontrol
Fungi	Tri	Connector	*Calvatia*	*Basidiomycota*	Coarse litter decomposition
Fungi	Tri	Connector	*Penicillium*	*Ascomycota*	Antifungal metabolism & P solubilization
Fungi	Tri	Connector	*Acremonium*	*Ascomycota*	Endophytic growth promotion
Fungi	Tri	Connector	*Blastobotrys*	*Ascomycota*	Oligotrophic C utilization
Fungi	Tri	Module hub	*Coniochaetales*	*Ascomycota*	Root symbiosis & OM decomposition
Fungi	Tri	Module hub	*Sordariomycetes*	*Ascomycota*	Multi-functional nutrient cycling

Note: Keystone taxa were identified based on Zi–Pi network analysis. Taxon represents the finest taxonomic resolutio n available for each keystone ZOTU, with genus as the primary classification level; taxa unclassified at the genus le

vel are annotated to the lowest identifiable rank. For connector taxa, only the five dominant genera within each treat ment are shown, ranked according to the cumulative relative abundance of their corresponding keystone ZOTUs. All i dentified module hubs and network hubs are included. Putative role denotes the inferred ecological function based o n published literature and known ecological characteristics of the corresponding taxa and should be interpreted as po tential rather than experimentally validated functions [55–60]. OM, organic matter; C, carbon; N, nitrogen; P, phosphor us. Connector, cross-module connecting taxa; Module hub, intra-module core taxa; Network hub, global keystone tax a. Identical genera were assigned consistent putative ecological roles across treatments.
